# Interaction between MARK3 (rs11623869), PLCB4 (rs6086746) and GEMIN2 (rs2277458) variants with bone mineral density and serum 25-hidroxivitamin D levels in Mexican Mestizo women

**DOI:** 10.3389/fendo.2024.1392063

**Published:** 2024-04-23

**Authors:** Diana I. Aparicio-Bautista, Rogelio F. Jiménez-Ortega, Adriana Becerra-Cervera, Arnoldo Aquino-Gálvez, Valeria Ponce de León-Suárez, Leonora Casas-Ávila, Jorge Salmerón, Alberto Hidalgo-Bravo, Berenice Rivera-Paredez, Rafael Velázquez-Cruz

**Affiliations:** ^1^ Laboratorio de Genómica del Metabolismo Óseo, Instituto Nacional de Medicina Genómica (INMEGEN), Mexico City, Mexico; ^2^ Departamento de Ciencias de la Acupuntura. Universidad Estatal del Valle de Ecatepec. Ecatepec de Morelos, Estado de Mexico, Mexico; ^3^ Consejo Nacional de Humanidades, Ciencias y Tecnologías (CONAHCYT), Mexico City, Mexico; ^4^ Laboratorio de Biología Molecular, Departamento de Fibrosis Pulmonar, Instituto Nacional de Enfermedades Respiratorias “Ismael Cosío Villegas”, Mexico City, Mexico; ^5^ Departamento de Medicina Genómica, Instituto Nacional de Rehabilitación, Mexico City, Mexico; ^6^ Centro de Investigación en Políticas, Población y Salud, Facultad de Medicina, Universidad Nacional Autónoma de México, Mexico City, Mexico

**Keywords:** osteoporosis, bone mineral density, vitamin D, genetic risk score, genetic association, postmenopausal

## Abstract

**Introduction:**

Understanding the genetic factors contributing to variations in bone mineral density (BMD) and vitamin D could provide valuable insights into the pathogenesis of osteoporosis. This study aimed to evaluate the association of single nucleotide variants in *MARK3* (rs11623869), *PLCB4* (rs6086746), and *GEMIN2* (rs2277458) with BMD in Mexican women.

**Methods:**

The gene-gene interaction was evaluated in these variants in serum 25(OH)D levels and BMD. A genetic risk score (GRS) was created on the basis of the three genetic variants. Genotyping was performed using predesigned TaqMan assays.

**Results:**

A significant association was found between the rs6086746-A variant and BMD at the total hip, femoral neck, and lumbar spine, in women aged 45 years or older. However, no association was observed between the variants rs11623869 and rs2277458. The rs11623869 × rs2277458 interaction was associated with total hip (*p*=0.002) and femoral neck BMD (*p*=0.013). Similarly, for vitamin D levels, we observed an interaction between the variants rs6086746 × rs2277458 (*p*=0.021). GRS revealed a significant association with total hip BMD (*p* trend=0.003) and femoral neck BMD (*p* trend=0.006), as well as increased vitamin D levels (*p* trend=0.0003). These findings provide evidence of the individual and joint effect of the *MARK3*, *PLCB4*, and *GEMIN2* variants on BMD and serum vitamin D levels in Mexican women.

**Discussion:**

This knowledge could help to elucidate the interaction mechanism between BMD-related genetic variants and 25OHD, contributing to the determination of the pathogenesis of osteoporosis and its potential implications during early interventions.

## 1 Introduction

Osteoporosis (OP) is a skeletal disease characterized by decreased bone mass and impaired microarchitecture leading to decreased mechanical strength and increased fracture risk (1). It has become a significant global public health concern due to the increasing number of fractures and the negative impact on the quality of life of affected individuals (2). Recent reports indicate that about 75 million people in Europe, the United States, and Japan are affected by OP, of which about 8.9 million have suffered fragility fractures. In Mexico, according to data from the 2020 population and housing census, the current population is around 126 million inhabitants, from which 17.4% correspond to individuals the subpopulation aged 50 years or older. It is estimated that about 10 million people in Mexico are living with OP (3, 4).

This disease is characterized by being multifactorial and complex where the predisposition, pathogenesis, or response to treatments are modulated by the interaction between genetic and environmental factors (5). The heritability of OP has been reported as high 50–85% (6, 7). The most effective approach for detecting the Single Nucleotide Variants (SNVs) associated to a multifactorial condition is through genome-wide association studies (GWAS). Therefore, several studies have aimed to link SNVs across the genome with the occurrence of OP, with the goal of identifying individuals at higher risk (8).

Microtubule affinity-regulating kinase 3 (*MARK3*) gene encodes a serine/threonine kinase, which is activated by the hepatic tumor suppressor kinase B1 (LKB1) and antagonizes oncogenic pathways, including the cell cycle pathway through phosphorylation of CDC25C (9). Regarding bone metabolism, a GWAS identified a locus containing multiple genes, including *MARK3*, *TRMT61*, and *CKB*. The main SNV associated was rs11623869, which lies in the second intron of *MARK3*. This locus was associated with femoral neck (FN) and lumbar spine (LS) BMD, and consistently replicated in Chinese and European populations (10, 11). A recent study implicates this kinase as an important signaling molecule in osteoblasts influencing bone mass (12).

Another study carried out in Taiwanese population identified the SNV rs6086746 upstream the Phosphoinositide Phospholipase C-Beta-4 (*PLCB4*) gene associated with low BMD in postmenopausal women. *PLCB4* encodes a phospholipase C which participates in the phosphoinositide cycle signaling pathway, transmitting information from the extracellular environment into the cell, influencing several cellular processes. It has been reported that rs6086746 may affect the binding of the transcription factor *RUNX2* to the promoter region of *PLCB4*, this might be part of the mechanisms contributing to the development of OP (13). *RUNX2* is a key transcription regulatory factor in osteoblast differentiation, it plays an important role in regulating osteoblast maturation and balance (14). Several studies reported that polymorphisms on the promoter of *RUNX2* are associated with BMD, in Korean and European populations (15–17).

The Gemin protein associated with the nuclear organelle 2 (GEMIN2) is part of a complex composed by the survival motor neuron protein (SMN) and seven additional Gemin proteins (*GEMIN2*–*8*). This complex is mainly involved in the assembly of the small nuclear ribonucleoprotein (snRNP) machinery, which regulates mRNA splicing in the cytoplasm (18). In particular, the SMN complex functions as a molecular chaperone whose phosphorylation regulates the biogenesis and function of snRNPs involved in mRNA splicing (19). Additionally, the SNV rs2277458 of the *GEMIN2* gene has been reported to be associated with the variation in plasma concentrations of 25-hydroxyvitamin D in the Danish population. In this population, low plasma concentration of 25-hydroxyvitamin D has been associated with a higher risk of osteoporotic fractures (20). Based on current evidence, this study aimed to investigate the effect of three recently identified SNVs (*MARK3-*rs11623869, *PLCB4-*rs6086746, and *GEMIN2*-rs2277458) on BMD, in a cohort of Mexican women. Additionally, we explored the effect of the interaction between these genetic variants with serum 25(OH)D levels and BMD.

## 2 Materials and methods

### 2.1 Study population

The study population included women born in Mexico whose parents and grandparents identified themselves as Mexican-mestizo. The population sample was composed of 1,300 middle-aged, unrelated women participating in the Health Workers Cohort Study (HWCS). The HWCS is a prospective study including workers from the Mexican Social Security Institute (IMSS) in Cuernavaca Morelos (central area of Mexico), focused on lifestyle and chronic diseases. Information on demographic characteristics, smoking status, menopausal status, medical history, and medication use was collected from each participant through a structured questionnaire (21). All study’s procedures were approved by the Ethics and Research Committee of the IMSS and all participants signed an informed consent form.

### 2.2 Bone mineral density measurement

Lumbar spine (L2-L4), femoral neck (FN), and total hip BMD were assessed using a Lunar DPX NT dual x-ray absorptiometry (DXA) instrument (Lunar Radiation Corp., Madison WI). Standard calibration of the instrument was performed daily using a phantom provided by the manufacturer for the femoral spine and neck. Technicians ensured that the daily coefficient of variation (CV) remained within normal operating standards and that the *in vivo* CV was less than 1.5%. BMD was calculated from bone mineral content (g) and bone area (cm^2^) to express it in g/cm^2^ and these data were used to analyze variations in BMD.

### 2.3 Single nucleotide variants genotyping and selection

A peripheral blood sample was taken from each patient and stored at 4°C until later use. Genomic DNA was extracted using a commercial isolation kit (QIAGEN System Inc., Valencia, CA), according to the manufacturer’s instructions. SNVs were selected from previous genome-wide association studies in the NCBI (www.ncbi.nlh.nih.gov/snp/) and Ensembl (http://asia.ensembl.org/) databases. Homo_sapiens/Info/Index). Genotyping of the rs11623869, rs6086746 and rs2277458 SNVs was performed using predesigned commercial TaqMan probes (Applied Biosystems, Foster City, CA, USA.) using a QuantStudio 7 Flex PCR system (Applied Biosystems, New Jersey, USA). Data were analyzed using Sequence Detection System (SDS) software, version 2.2.1.

### 2.4 Statistical analysis

Data from the study population are shown as median for quantitative variables and absolute and relative frequencies for qualitative variables. Hardy-Weinberg equilibrium was conducted for each SNV using the standard χ^2^ test, which is a fundamental analysis in population genetics to assess whether a population is evolving at a neutral state. This test helps ensure the reliability of genetic association studies by evaluating the expected and observed genotype frequencies within a population. Linear and logistic regression analysis were used to test the association between BMD, serum 25(OH)D levels, and genotype. Both continuous and categorical measurements of BMD and serum 25(OH)D levels were considered in the analysis, as appropriate. Codominant, additive, recessive, and dominant genetic models were used. The BMD models were adjusted for age (years), BMI categories, energy intake, calcium intake (tertiles), vitamin D intake (tertiles), calcium supplementation, alcohol consumption (g/day), smoking status (never, current and past), physical activity, and hormone replacement therapy (HRT). The vitamin D models were adjusted for age (years), BMI categories, energy intake, vitamin D intake (tertiles), alcohol consumption (g/day), smoking status (never, current and past), physical activity, blood collection season, and HRT. To explore potential gene-gene interactions, we incorporated a term for gene-gene interaction into the statistical models. This allowed us to assess how the effects of one gene may modify or influence the effects of another gene within the studied population. By examining these interactions, we aimed to gain deeper insights into the complex interplay between genetic factors and their combined impact on the outcome of interest. We estimated the genetic risk score by summing the risk alleles of the three genetic variants. We collapsed women with 4 and 5 risk alleles into the category of 3 alleles due to their low frequency. For all statistical tests, we used Statistical Software for Data Science version 18 (STATA v18.0, TX, USA.). Values of *p*< 0.05 were considered statistically significant.

## 3 Results

### 3.1 Population characteristics- HWCS

For the present study, a total of 1,300 females were included. The median age of the study sample was 54 years (P25-P75, 43-63). According to the body mass index, 39.9% were overweight and 26.2% were obese. The median total hip BMD was 0.964 g/cm^2^ (0.871-1.072), and 27.9% had low total hip BMD. The median femoral neck BMD was 0.932 g/cm^2^ (0.830-1.027), and 42.1% had low femoral neck BMD. The median lumbar spine BMD was 1.068 g/cm^2^ (0.950-1.174), and 53.1% had low lumbar spine BMD (**Supplementary Table 1**).

### 3.2 Minor allele frequency of SNVs

The distributions of the alleles of the three SNVs were analyzed by Hardy–Weinberg equilibrium in the HWCS. The variants demonstrated Hardy-Weinberg equilibrium, with *p-*values of 0.36 for rs1050450, 0.38 for rs6086746, and 0.72 for rs11623869. The MAFs of the three SNVs differ from the reported for CEU population. However, were similar to data reported for the Mexican Ancestry population living in Los Angeles, CA, USA (MXL) (Data not shown).

### 3.3 Association analyses between the SNVs and bone mineral density (g/cm^2^), and low-BMD

Anthropometric and biochemical characteristics of the study population based on rs11623869, rs6086746, and rs2277458 genotypes are presented in **Supplementary Tables 2**–**4**, respectively. Women carrying at least one copy of the T allele of rs11623869 had a higher median of LDL levels, and a higher prevalence of elevated LDL. In addition, these women also had a higher prevalence of low BMD at the total hip and femoral neck. No other statistically significant differences were observed (**Supplementary Table 2**). On the other hand, women carrying at least one copy of the A allele of rs6086746 had a lower median fasting glucose level, lower triglyceride levels, and lower prevalence of elevated triglycerides compared to women homozygous for the ancestral allele. Furthermore, women carrying at least one copy of the A allele had a lower prevalence of low BMD, at the femoral neck, total hip and lumbar spine (**Supplementary Table 3**). We did not observe statistically significant differences for the genotypes of the SNV rs2277458 variant (**Supplementary Table 4**).

Afterwards, we looked for association of the SNVs with BMD and levels of 25-hydroxivitamin D. Association analysis was conducted through adjusted logistic regression models. The adjusted models revealed that rs11623869 and rs2277458 were not associated with BMD at the analyzed sited. In contrast, the rs6086746 variant, under different inheritance models, showed that the A allele was associated with higher values of BMD at the total hip, femoral neck, and lumbar spine compared to the G allele (**Table 1**). Consistent associations were observed in women aged 45 years or older, where the A allele of variant rs6086746 showed a significant association with higher BMD at the total hip, femoral neck, and lumbar spine, compared to the G allele (**Table 2**). In contrast, no significant associations were observed in women younger than 45 years old (Data not shown).

**Table 1:** Association between the SNVs, BMD at different sites, and 25-hydroxivitamin D levels in total women.

		Total hip BMD		Femoral neck BMD		Lumbar spine BMD		25-hydroxivitamin D	
rs11623869		β (95%CI)	*p*-value	β (95%CI)	*p*-value	β (95%CI)	*p*-value	β (95%CI)*	*p*-value
Additive		-0.0001 (-0.012,0.012)	0.982	0.003 (-0.009,0.014)	0.662	0.002 (-0.013,0.016)	0.834	0.76 (0.12,1.40)	0.021
Codominant	GG	0.0		0.0		0.0		0.0	
	GT	0.004 (-0.010,0.018)	0.577	0.004 (-0.010,0.017)	0.579	0.008 (-0.010,0.025)	0.382	0.95 (0.18,1.72)	0.016
	TT	-0.015 (-0.051,0.021)	0.412	-0.001 (-0.034,0.035)	0.983	-0.019 (-0.064,0.026)	0.398	0.84 (-1.16,2.84)	0.411
Dominant	GG	0.0		0.0		0.0		0.0	
	GT+TT	0.002 (-0.011,0.016)	0.752	0.003 (-0.010,0.016)	0.600	0.005 (-0.012,0.022)	0.543	0.94 (0.19,1.68)	0.013
Recessive	GG+GT	0.0		0.0		0.0		0.0	
	TT	-0.005 (-0.052,0.019)	0.366	-0.0009 (-0.035,0.034)	0.958	-0.022 (-0.067,0.023)	0.332	0.51 (-1.47,2.50)	0.611
rs6086746									
Additive		0.020 (0.010,0.031)	0.0002	0.019 (0.008,0.029)	0.0004	0.028 (0.015,0.042)	0.00003	0.37 (-0.22,0.96)	0.224
Codominant	GG	0.0		0.0		0.0		0.0	
	GA	0.020 (0.007,0.033)	0.004	0.018 (0.005,0.031)	0.007	0.034 (0.017,0.051)	0.00006	0.68 (-0.06,1.42)	0.072
	AA	0.042 (0.014,0.070)	0.003	0.039 (0.012,0.066)	0.005	0.043 (0.008,0.078)	0.016	0.04 (-1.50,1.58)	0.963
Dominant	GG	0.0		0.0		0.0		0.0	
	GA+AA	0.023 (0.010,0.034)	0.001	0.021 (0.008,0.033)	0.001	0.035 (0.019,0.051)	0.00002	0.60 (-0.12,1.32)	0.101
Recessive	GG+GA	0.0		0.0		0.0		0.0	
	AA	0.033 (0.006,0.060)	0.017	0.031 (0.004,0.057)	0.023	0.028 (-0.006,0.063)	0.110	-0.26 (-1.77,1.24)	0.731
rs2277458									
Additive		-0.003 (-0.013,0.009)	0.631	-0.0003 (-0.010,0.010)	0.946	-0.004 (-0.017,0.009)	0.591	0.78 (0.21,1.35)	0.008
Codominant	GG	0.0		0.0		0.0		0.0	
	GA	-0.003 (-0.016,0.011)	0.702	-0.003 (-0.016,0.010)	0.694	-0.008 (-0.024,0.009)	0.374	0.74 (0.001,1.47)	0.050
	AA	-0.005 (-0.032,0.022)	0.716	0.004 (-0.022,0.030)	0.775	0.0009 (-0.033,0.034)	0.959	1.63 (0.16,3.10)	0.029
Dominant	GG	0.0		0.0		0.0		0.0	
	GA+AA	-0.003 (-0.016,0.010)	0.653	-0.002 (-0.014,0.011)	0.788	-0.006 (-0.022,0.010)	0.434	0.86 (0.16,1.57)	0.017
Recessive	GG+GA	0.0		0.0		0.0		0.0	
	AA	-0.004 (-0.030,0.022)	0.772	0.005 (-0.020,0.030)	0.706	0.004 (-0.029,0.037)	0.807	1.32 (-0.11,2.76)	0.071

Model adjusted for age (years), BMI categories, energy intake, calcium intake (tertiles), vitamin D intake (tertiles), calcium supplementation, alcohol consumption (g/day), smoking status (never, current and past), physical activity, and hormone replacement therapy (HRT). *Model adjusted for age (years), BMI categories, energy intake, vitamin D intake (tertiles), alcohol consumption (g/day), smoking status (never, current and past), physical activity, blood collection season, and hormone replacement therapy (HRT).

**Table 2:** Association between the SNVs and BMD at different sites and 25-hydroxivitamin D levels among women aged 45 years and older.

		Total hip BMD		Femoral neck BMD		Lumbar spine BMD		25-hydroxivitamin D	
rs11623869		β (95%CI)	*p*-value	β (95%CI)	*p*-value	β (95%CI)	*p*-value	β (95%CI)*	*p*-value
Additive		0.004 (-0.009,0.018)	0.556	0.005 (-0.008,0.018)		0.003 (-0.015,0.021)	0.741	0.78 (0.03,1.54)	0.041
Codominant	GG	0.0		0.0		0.0		0.0	
	GT	0.007 (-0.009,0.023)	0.400	0.007 (-0.008,0.023)	0.352	0.007 (-0.014,0.028)	0.524	0.91 (0.02,1.80)	0.045
	TT	-0.003 (-0.045,0.040)	0.906	0.001 (-0.039,0.042)	0.945	-0.009 (-0.064,0.047)	0.762	1.09 (-1.25,3.44)	0.361
Dominant	GG	0.0		0.0		0.0		0.0	
	GT+TT	0.006 (-0.010,0.022)	0.446	0.007 (-0.008,0.022)	0.373	0.005 (-0.015,0.026)	0.600	0.93 (0.06,1.79)	0.036
Recessive	GG+GT	0.0		0.0		0.0		0.0	
	TT	-0.005 (-0.047,0.037)	0.817	-0.001 (-0.041,0.039)	0.957	-0.011 (-0.066,0.044)	0.696	0.78 (-1.55,3.11)	0.510
rs6086746									
Additive		0.023 (0.011,0.036)	0.00002	0.020 (0.008,0.031)	0.001	0.034 (0.018,0.051)	0.00004	0.69 (-0.006,1.39)	0.052
Codominant	GG	0.0		0.0		0.0		0.0	
	GA	0.025 (0.009,0.041)	0.002	0.022 (0.007,0.037)	0.005	0.044 (0.023,0.064)	0.00003	1.17 (0.29,2.05)	0.009
	AA	0.042 (0.010,0.075)	0.011	0.034 (0.003,0.066)	0.031	0.048 (0.005,0.091)	0.028	0.34 (-1.48,2.17)	0.713
Dominant	GG	0.0		0.0		0.0		0.0	
	GA+AA	0.027 (0.012,0.042)	0.0004	0.023 (0.009,0.038)	0.002	0.044 (0.024,0.064)	0.00001	1.06 (0.22,1.91)	0.014
Recessive	GG+GA	0.0		0.0		0.0		0.0	
	AA	0.031(-0.0005,0.063)	0.054	0.025(-0.006,0.056)	0.109	0.029 (-0.013,0.072)	0.173	-0.17 (-1.96,1.62)	0.851
rs2277458									
Additive		0.001 (-0.011,0.013)	0.862	0.002 (-0.010,0.013)	0.768	-0.003 (-0.019,0.013)	0.727	0.96 (0.29,1.64)	0.005
Codominant	GG	0.0		0.0		0.0		0.0	
	GA	-0.0002 (-0.016,0.015)	0.977	-0.003 (-0.018,0.012)	0.678	-0.006 (-0.026,0.014)	0.563	0.87 (0.01,1.74)	0.048
	AA	0.005 (-0.026,0.036)	0.765	0.013 (-0.017,0.043)	0.382	0.0007 (-0.040,0.041)	0.972	2.11 (0.38,3.85)	0.017
Dominant	GG	0.0		0.0		0.0		0.0	
	GA+AA	0.0005 (-0.014,0.015)	0.951	-0.0009 (-0.015,0.013)	0.906	-0.005 (-0.025,0.014)	0.611	1.04 (0.21,1.87)	0.014
Recessive	GG+GA	0.0		0.0		0.0		0.0	
	AA	0.005 (-0.026,0.035)	0.755	0.015 (-0.015,0.044)	0.325	0.003 (-0.037,0.043)	0.873	1.73 (0.05,3.46)	0.044

Model adjusted for age (years), BMI categories, energy intake, calcium intake (tertiles), vitamin D intake (tertiles), calcium supplementation, alcohol consumption (g/day), smoking status (never, current and past), physical activity, and hormone replacement therapy (HRT). *Model adjusted for age (years), BMI categories, energy intake, vitamin D intake (tertiles), alcohol consumption (g/day), smoking status (never, current and past), physical activity, blood collection season, and hormone replacement therapy (HRT).

In the adjusted additive, codominant, and dominant models, only the A allele of the rs6086746 variant showed a protective effect for low BMD at various sites (**Table 3**, **Supplementary Table 5**). These associations showed a reduction of the odds of having low BMD of approximately 33-55% at the sites analyzed.

**Table 3:** Association between the variants of interest and low-BMD at different sites and VD deficiency in total women.

		Total hip BMD		Femoral neck BMD		Lumbar spine BMD		VD Deficiency*	
rs11623869		OR (95%CI)	*p*-value	OR (95%CI)	*p*-value	OR (95%CI)	*p*-value	OR (95%CI)	*p*-value
Additive		1.18 (0.91-1.54)	0.220	1.13 (0.89-1.45)	0.321	0.96 (0.76-1.21)	0.731	0.84 (0.68-1.04)	0.118
Codominant	GG	1.0		1.0		1.0		1.0	
	GT	1.39 (1.01-1.90)	0.042	1.21 (0.90-1.62)	0.212	0.88 (0.67-1.15)	0.346	0.79 (0.61-1.02)	0.069
	TT	0.77 (0.32-1.82)	0.547	1.03 (0.48-2.20)	0.936	1.29 (0.63-2.63)	0.491	0.91 (0.47-1.75)	0.766
Dominant	GG	1.0		1.0		1.0		1.0	
	GT+TT	1.31 (0.97-1.79)	0.080	1.19 (0.89-1.58)	0.236	0.91 (0.70-1.18)	0.478	0.80 (0.63-1.02)	0.076
Recessive	GG+GT	1.0		1.0		1.0		1.0	
	TT	0.68 (0.29-1.61)	0.383	0.97 (0.46-2.04)	0.928	1.35 (0.66-2.73)	0.411	0.98 (0.51-1.88)	0.951
rs6086746									
Additive		0.74 (0.57-0.95)	0.020	0.68 (0.54-0.86)	0.001	0.71 (0.57-0.86)	0.001	0.91 (0.75-1.10)	0.329
Codominant	GG	1.0		1.0		1.0		1.0	
	GA	0.69 (0.50-0.95)	0.021	0.68 (0.50-0.91)	0.009	0.65 (0.50-0.86)	0.002	0.85 (0.67-1.09)	0.198
	AA	0.65 (0.33-1.29)	0.217	0.47 (0.25-0.88)	0.018	0.60 (0.34-1.04)	0.070	0.95 (0.57-1.57)	0.843
Dominant	GG	1.0		1.0		1.0		1.0	
	GA+AA	0.69 (0.51-0.93)	0.015	0.65 (0.49-0.86)	0.002	0.65 (0.50-0.84)	0.001	0.86 (0.68-1.09)	0.222
Recessive	GG+GA	1.0		1.0		1.0		1.0	
	AA	0.76 (0.39-1.48)	0.419	0.55 (0.30-1.02)	0.060	0.72 (0.42-1.24)	0.236	1.02 (0.62-1.66)	0.940
rs2277458									
Additive		1.11 (0.87-1.41)	0.409	1.08 (0.87-1.32)	0.494	0.87 (0.69-1.09)	0.219	0.90 (0.74-1.08)	0.253
Codominant	GG	1.0		1.0		1.0		1.0	
	GA	1.06 (0.78-1.46)	0.695	1.06 (0.81-1.38)	0.674	0.95 (0.71-1.26)	0.708	0.90 (0.71-1.14)	0.385
	AA	1.32 (0.72-2.42)	0.372	1.19 (0.70-2.04)	0.519	0.63 (0.35-1.14)	0.126	0.80 (0.49-1.30)	0.365
Dominant	GG	1.0		1.0		1.0		1.0	
	GA+AA	1.12 (0.83-1.50)	0.472	1.08 (0.83-1.39)	0.573	0.90 (0.68-1.18)	0.437	0.88 (0.70-1.11)	0.296
Recessive	GG+GA	1.0		1.0		1.0		1.0	
	AA	1.38 (0.77-2.46)	0.277	1.16 (0.69-1.97)	0.570	0.65 (0.36-1.15)	0.138	0.83 (0.51-1.35)	0.457

Model adjusted for age (years), BMI categories, energy intake, calcium intake (tertiles), vitamin D intake (tertiles), calcium supplementation, alcohol consumption (g/day), smoking status (never, current and past), physical activity, and hormone replacement therapy (HRT). Low-BMD as a T-score below -1 at the total hip, and lumbar spine. *Model adjusted for age (years), BMI categories, energy intake, vitamin D intake (tertiles), alcohol consumption (g/day), smoking status (never, current and past), physical activity, blood collection season, and hormone replacement therapy (HRT).

### 3.4 Association analyses between the SNVs and serum 25-hydroxyvitamin D levels

The variants rs11623869 and rs2277458 were associated with higher levels of vitamin D. Under the dominant model, having at least one copy of the T allele of variant rs11623869 and at least one copy of the A allele of variant rs2277458 was associated with higher serum vitamin D levels compared to women carrying the wild-type allele (β= 0.94, 95% CI 0.19-1.68 and β = 0.86, 95% CI 0.16-1.57, respectively). However, variant rs6086746 did not show a statistically significant association (**Table 1**). Stratified analysis by age groups revealed a similar association only in women aged 45 years or older (**Supplementary Table 5**, **Table 2**). The associations for vitamin D deficiency were not statistically significant in either the total women or the age-stratified analysis (**Table 3**, **Supplementary Table 5**).

### 3.5 Interaction between SNVs with bone mineral density

**Figure 1:**
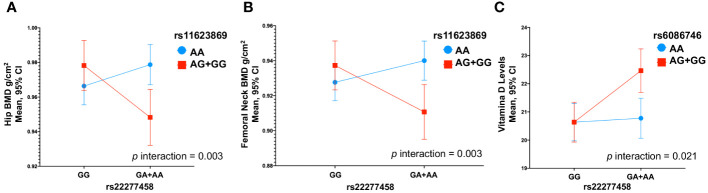
Gene-Gene Interactions Impacting BMD and 25(OH)D Levels in Total Women. **(A)** rs11623869 × rs2277458 interaction with total hip, **(B)** rs11623869 × rs2277458 interaction with femoral neck, and **(C)** rs6086746 × rs2277458 interaction with vitamin D levels. Model **(A, B)** adjusted for age (years), BMI categories, energy intake, calcium intake (tertiles), vitamin D intake (tertiles), calcium supplementation, alcohol consumption (g/day), smoking status (never, current, and past), physical activity, and hormone replacement therapy (HRT). Low-BMD as a T-score below -1 at the total hip, and lumbar spine. Model **(C)** adjusted for age (years), BMI categories, energy intake, vitamin D intake (tertiles), alcohol consumption (g/day), smoking status (never, current, and past), physical activity, blood collection season, and hormone replacement therapy (HRT).

**Figure 2:**
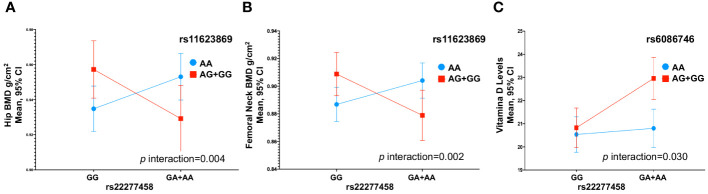
Gene-Gene Interactions Affecting BMD and 25(OH)D Levels in Women Aged > 45 Years. **(A)** rs11623869 × rs2277458 interaction with total hip, **(B)** rs11623869 × rs2277458 interaction with femoral neck, and **(C)** rs6086746 × rs2277458 interaction with vitamin D levels. Model **(A, B)** adjusted for age (years), BMI categories, energy intake, calcium intake (tertiles), vitamin D intake (tertiles), calcium supplementation, alcohol consumption (g/day), smoking status (never, current, and past), physical activity, and hormone replacement therapy (HRT). Low-BMD as a T-score below -1 at the total hip, and lumbar spine. Model **(C)** adjusted for age (years), BMI categories, energy intake, vitamin D intake (tertiles), alcohol consumption (g/day), smoking status (never, current, and past), physical activity, blood collection season, and hormone replacement therapy (HRT).

We observed an interaction between the variants rs11623869 × rs2277458 with total hip (*p* interaction=0.002) and femoral neck BMD (*p* interaction=0.013). In carriers with at least one copy of the G allele of variant rs11623869 and at least one copy of the A allele of variant rs2277458, lower BMD was observed in the total hip (β= -0.030, 95%CI -0.052, -0.008, *p*=0.007) and femoral neck (β= -0.026, 95%CI -0.048, -0.006, *p*=0.013), compared to carriers of the wild-type allele of variant rs2277458. While carriers of the wild-type allele of variant rs11623869 showed no significant differences, carriers with at least one copy of the A allele of variant rs2277458 demonstrated lower BMD at the total hip (β=0.012, 95%CI -0.004, 0.028, *p*=0.128) and femoral neck (β=0.012, 95%CI -0.003, 0.028, *p*=0.115) (**Figures 1A, B**). These patterns were observed in women aged ≥45 years (**Figures 2A, B**) but not in women aged <45 years (*p* interaction total hip=0.623 and *p* interaction femoral neck=0.827). No significant interactions were observed with lumbar spine BMD.

### 3.6 Interaction between SNVs with serum 25-hydroxivitamin D levels

Similarly, for vitamin D levels, we found a distinct interaction between the variants rs6086746 × rs2277458 (*p* interaction=0.021). Carriers with at least one copy of the A allele of variant rs6086746 and at least one copy of the A allele of variant rs2277458 exhibited higher vitamin D levels (β=1.83, 95%CI 0.78, 2.88, *p*=0.001). However, this is not significant with respect to individuals carrying the wild-type allele of the rs2277458 variant. Conversely, in carriers of at least one copy of the G allele of variant rs2277458 association was not statistically significant (β= 0.14, 95%CI -0.83, 1.11, *p*=0.776), when rs6086746 was of the wild-type (**Figure 1C**). These patterns were consistently observed in women aged ≥45 years (*p*=0.030) (**Figure 2C**) but not in women aged <45 years (*p* interaction=0.672).

### 3.7 Genetic risk score

**Figure 3:**
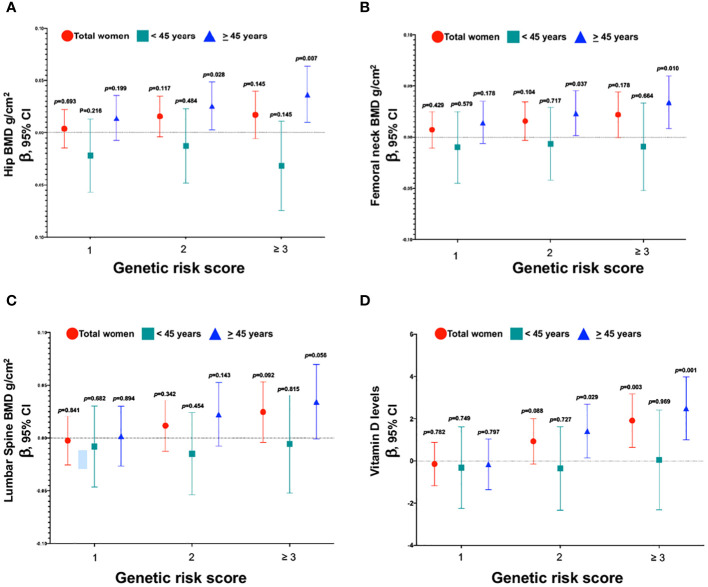
Association between the genetic risk score of variants with BMD and vitamin D levels. **(A)** Genetic risk score and total hip, **(B)** Genetic risk score and femoral neck, **(C)** Genetic risk score and lumbar spine, and **(D)** Genetic risk score and vitamin D levels. Model **(A–C)** were adjusted for age (years), BMI categories, energy intake, calcium intake (tertiles), vitamin D intake (tertiles), calcium supplementation, alcohol consumption (g/day), smoking status (never, current, and past), physical activity, and hormone replacement therapy (HRT). Low-BMD as a T-score below -1 at the total hip, and lumbar spine. Model **(D)** was adjusted for age (years), BMI categories, energy intake, vitamin D intake (tertiles), alcohol consumption (g/day), smoking status (never, current, and past), physical activity, blood collection season, and hormone replacement therapy (HRT).

We created a risk score based on the number of risk alleles across the three genetic variants. Since rs11623869 and rs2277458 were statistically associated with higher vitamin D levels, and rs6086746 showed a borderline association, we clustered together the categories of 3, 4, and 5 risk alleles due to their low frequency. Among women aged ≥45 years, those carrying ≥3 risk alleles had, on average, higher total hip (*p* trend=0.003) and femoral neck BMD (*p* trend=0.006), as well as elevated serum 25(OH) D levels (*p* trend=0.00003), compared to those carrying 0 risk alleles (**Figures 3A, B, D**). However, this association was not statistically significant for lumbar spine BMD (**Figure 3C**). Notably, we observed an association solely between the risk score and vitamin D levels for the overall cohort of women, but not for BMD. Furthermore, in women aged <45 years, no significant associations were identified.

## 4 Discussion

Our study confirmed previous evidence indicating associations between the genetic variants *MARK3* (rs11623869), *PLCB4* (rs6086746), and *GEMIN2* (rs2277458) with osteoporosis and serum 25OHD levels in a European-descent population and a southern Chinese cohort also associated with an admixed population such as the Mexican-Mestizo. These findings suggest that the genetic factors influencing osteoporosis and vitamin D levels may transcend ethnic boundaries, emphasizing their relevance across diverse population groups.

In recent years, it has been reported that circulating factors such as calcium, phosphate, 25(OH)D, PTH and ALP, have been related to the variation of DMO. They can act directly or indirectly on skeletal cells, regulating bone remodeling and metabolism. Vitamin D is essential for efficient calcium absorption. An adequate amount of vitamin D is required to maintain bone strength and prevent fragility fractures (22) Vitamin D deficiency (25OHD <20 ng/mL) is common in Mexican older adults and linked with factors such as sex, age, genetics, diet and obesity (23, 24). In addition, the lower intake of vitamin D and calcium reported in the Mexican population (25) can cause inefficient absorption of calcium, which in turn can stimulate the release of calcium from the bones to maintain a normal concentration of calcium in the blood and consequently cause bone loss. Decreasing serum calcium levels can stimulate PTH secretion, and PTH in turn can improve serum calcium concentration by releasing calcium from bone by increasing bone resorption. However, several studies have reported the negative impact of elevated serum PTH levels on BMD (26). Calcium and phosphate are co-dependent for bone development, a sufficient amount of phosphate in the blood has a positive effect on calcium use and adequate bone growth (27), while ALP plays an important role in the formation and mineralization of osteoid and is used as a biomarker to evaluate bone turnover. The high rate of bone turnover in elderly people can lead to rapid bone loss and reduced bone mass. Interactions between genetic, dietary, hormonal, metabolic, and lifestyle factors have been suggested to play an important role in susceptibility to low BMD. In this sense, the risk caused by genetic variants may vary between individual’s due to differential modifications of the different concentrations of circulating factors involved in bone remodeling and metabolism. While a study by Xiao SM, et al. in 2013 reported an association between the SNV rs11623869 of the MARK3 gene and BMD, particularly strengthened in the presence of high serum levels of ALP (22), no association studies have been conducted for the SNVs rs6086746 in PLCB4 and rs2277458 in GEMIN2 with serum variables such as vitamin D, calcium or ALP, and their potential effects on BMD variation.

It has been reported that the *MARK3* gene is involved in different biological processes such as cell cycle, ciliated cell differentiation, and osteoclast differentiation (28). So far, the role of *MARK3* has been studied in several pathological conditions (9, 29–31). In osteoporosis, the variant rs11623869 in *MARK3* has been associated with bone mineral density and low-trauma fractures (32, 33). Our results showed that the rs11623869-T variant is associated with low BMD at the total hip. These data are consistent with previous reports where the T allele was associated with a decreased BMD at femoral neck accompanied by an increased expression of *MARK3* (32). The authors showed that *Mark3*-deficient osteoblasts exhibited an increase of bone mass, through reduced *Jag1/Hes1* expression and decreased downstream JNK signaling stimulating osteoblast activity. On the other hand, in Chinese population, it was observed that the effect of the SNV rs11623869 on BMD was greater in the presence of high serum levels of ALP, a biomarker for osteoblast activity, and considered a predictor of BMD in postmenopausal females (33). Together these data suggest that genetic variation of *MARK3* influences bone mineral density. However, current data also suggest that circulating factors in serum may also affect bone turnover and metabolism modifying the association of *MARK3* with BMD. Further research is necessary to clarify the role of these circulatory factors in Mexican postmenopausal women.

Another gene of interest is *PLCB4*, its product catalyzes the formation of inositol 1,4,5-trisphosphate and diacylglycerol from phosphatidylinositol 4,5-bisphosphate, using calcium as a cofactor. *PLCB4* plays an essential role in signal transduction (34). Genetic variants in *PLCB4* have been identified as cause of the Auriculocondylar syndrome 2 (ARCND2), a disease characterized by craniofacial malformations (13), *PLCB4* acts as a direct signaling effector of the endothelin receptor type A (EDNRA)-Gq/11 pathway. Kanai et al, 2022 demonstrated that variants in *PLCB4* gene interfere with the EDNRA signaling pathway, leading to the development of ARCND2 (35). However, the role of genetic variants in *PLCB4* on BMD has been explored only to a limited extent. Only one study in the Chinese population reported that carriers of the A alleles of the rs6086746 variant in *PLCB4*, showed a decrease in BMD and an increased risk of developing osteoporosis. Furthermore, they found that the rs6086746 variant was significantly associated with osteoporosis, by participating on the binding of *RUNX2*, a master transcription factor involved in the osteoblast maturation. Through luciferase assays, they showed increased to that *PLCB4* activity in individuals carrying the rs6086746-A allele, proposing a functional explanation for the observed association (13). In contrast, our results shown that variant rs6086746 was associated with higher BMD and protective effect against low-BMD in women aged 45 years or older. These results are consistent with an independent group of OP patients from the National Institute of Rehabilitation (INR) in Mexico City (36). Although the frequencies were similar in both cohorts, we did not observe statistical differences (OR = 0.74, 95%CI 0.47-1.16), perhaps due to the small sample size (n=384).

Recently, three genotypes associated to plasma 25-hydroxyvitamin D levels were identified through genome-wide association studies; among which is the variant rs2277458 in *GEMIN2* (37). GEMIN2 encodes a protein of the SMN complex, this complex includes several Gemin proteins and the SMN protein. The SMN complex is located at a subnuclear compartment called gems (Gemin of coiled bodies) and is necessary for the assembly of spliceosomal snRNPs and for pre-mRNA splicing. Although there is no evidence implicating *GEMIN2* in vitamin-D-related physiological pathways.

More recently, in a study in the Danish population, the rs2277458-A allele of *GEMIN2* was associated with lower serum 25-hydroxyvitamin D concentrations in a dose-dependent manner (20). These data are consistent with our results where the rs2277458-G allele of *GEMIN2* was associated with lower serum 25-hydroxyvitamin D levels, in women aged 45 years or older. Low vitamin D concentrations are believed to affect bone metabolism by decreasing dietary calcium and phosphorus absorption and increasing the production of parathyroid hormone (38). In addition, vitamin D activates osteoblasts and osteoclasts to dissolve the mineralized collagen in bones, causing osteopenia and osteoporosis, thus increasing the risk of fractures. Together these data suggest that genetic variants in *GEMIN2* influences serum 25-hydroxyvitamin D concentrations, in different populations (39).

Vitamin D has been shown to play a fundamental role in calcium and phosphate homeostasis, furthermore, serum levels of vitamin D are positively correlated with BMD values (40). In contrast, vitamin D deficiency causes a reduction of BMD, increasing the risk of bone fractures in the elderly (41). In our study, the genetic risk score constructed using the three variants (equivalent to 3 risk alleles) was significantly associated with higher BMD at the hip and femoral neck, as well as elevated serum 25OHD levels. These associations were particularly pronounced among postmenopausal women aged 45 years and older. This suggests that the cumulative genetic risk, as represented by the GRS, may contribute to improved bone health and vitamin D status in this population. These results are consistent with those reported by Mithal, et al., 2009, who analyzed a population from Latin America including Mexico that was composed of postmenopausal women and in which they observed that vitamin D levels are below of the average values, due to geographical and population characteristics (23, 42).

A possible explanation for these findings is that vitamin D consumption could improve BMD levels in women over 45 years of age with higher genetic risk, given that the associated genetic variants are linked to vitamin D levels in the body. Additionally, the SNVs included in the genetic risk score may influence vitamin D metabolism, thereby strengthening the response. Therefore, these data may generate a new focus on the possible role of these SNVs on vitamin D metabolism. Nevertheless, these data should be taken with caution, as a larger and more diverse population is needed for confirming or discarding these findings.

The literature regarding the interaction between genes related to vitamin D levels and BMD, in Mexican Mestizo population is scarce (43). This study provides evidence suggesting that this phenomenon could be participating in the high prevalence of vitamin D deficiency in the Mexican population. Furthermore, interactions between genes are proposed to serve as a key factor involved in the variance of BMD. We have identified an effect of the interaction between SNV rs11623869 in the *MARK3* gene and the SNV rs22277458 in *GEMIN2* on hip and femoral neck BMD. The influence of these interactions on BMD is complex, concentrations of circulating factors related to calcium and phosphate metabolism might alter the effect of genetic risk factors and the subsequent loss of BMD. Studies considering the interaction of genes involved in BMD loss in conjunction with known factors involved in calcium and phosphorus metabolism will contribute to unravel this complex relationship. Furthermore, interactions between genetic and lifestyle factors have been suggested to play an important role on susceptibility to having low BMD and serum 25OHD levels. The risk caused by genetic variants may vary between populations due to exposition to different environmental factor and lifestyle habits. In this study, we found that the association of the rs22277458 in *GEMIN2* with serum 25OHD levels was strengthened in the presence of the rs6086746 *PCLB4*. However, the role of *GEMIN2* and *PCLB4* on vitamin D metabolism remains unknown. Also, we cannot exclude that variants in these genes may only indirectly affect 25 hydroxyvitamin D through pathways affecting both, exposure and outcome separately. Further, we cannot exclude the possibility that other mechanisms involving these genes may exist. Finally, the association of genetic variants in *GEMIN2* and *PCLB4* genes with serum 25OHD could represent a chance finding and, therefore, needs additional confirmation in an independent cohort.

This study has some strengths: first this analysis was conducted in a relatively large sample (n=1300) compared to other observational studies. Second, this is the first study focused on understanding the effect of genetic variants involved in vitamin D metabolism and bone mineral density in postmenopausal women. On the other hand, this study has some limitations. First, this work did not analyze GRS with serum calcium levels and bone fracture as reported in other studies. This could strengthen our hypothesis about the effect of SNVs of genes involved in vitamin D metabolism. Furthermore, since our study is cross-sectional, we cannot establish causality between SNVs, vitamin D levels, and BMD. In this work, it will be observed that vitamin D consumption is positively associated with BMD and is essential for efficient calcium absorption. However, a limitation of this study is that the relationship between serum calcium, ALP, and phosphate with BMD was not analyzed, these variables that have previously been reported for their association with bone growth and adequate osteoid mineralization. Furthermore, we acknowledge that the interaction observed between MARK3 and vitamin D levels may require replications in other independent populations, and functional studies are necessary to investigate whether the effect of MARK3 on BMD is regulated by vitamin D levels. Another limitation is that our study represents the first report showing the association between the SNVs rs6086746 in PLCB4, and rs2277458 in GEMIN2 with vitamin D levels and BMD in a Mexican population, as these genes and their respective variants had not been previously related to bone metabolism, and there are no reports on their functional role. In addition to these limitations, show the need for future longitudinal research that can address these issues. Second, we did not adjust for multiple comparisons due to the effect size found, although in recent years it has been reported that adjustment by multiple testing controls overall type I error but significantly increases type II error (44).

## 5 Conclusions

Our study has provided independent replications of the associations reported for the *MARK3* (rs11623869), *PLCB4* (rs6086746), and *GEMIN2* (rs2277458) genetic variants with BMD and serum 25 hydroxy-vitamin D in a Mexican mestizo population. These results suggest that that genetic variants in these three genes may confer susceptibility for changes in BMD and serum 25 hydroxy vitamin D levels in Mexican-Mestizo, Chinese, and European-descent populations. Furthermore, we have found that SNVs rs22277458 in *GEMIN2*, rs11623869 in *MARK3* and rs6086746 in *PLCB4*, highlighting the complexity of genetic and environmental factors in determining bone health. These findings have important clinical and public health implications as they could help improve the prevention, diagnosis, and treatment of bone diseases such as osteoporosis in the Mexican population. However, further longitudinal research is required to fully understand the underlying mechanisms and clinical implications of these genetic associations.

## Data availability statement

The original contributions presented in the study are included in the article/supplementary material, further inquiries can be directed to the corresponding author.

## Ethics statement

The studies involving humans were approved by Ethics Committee from Mexican Social Security Institute (No. 12CEI 09 006 14), and the National Institute of Genomic Medicine (399–17/2016/I). The studies were conducted in accordance with the local legislation and institutional requirements. The participants provided their written informed consent to participate in this study.

## Author contributions

DA-B: Writing – original draft, Writing – review & editing, Investigation, Visualization. RJ-O: Investigation, Visualization, Writing – original draft, Writing – review & editing. AB-C: Writing – review & editing, Methodology. AA-G: Writing – review & editing, Funding acquisition. VL-S: Writing – review & editing, Methodology. LC-A: Methodology, Writing – review & editing. JS: Funding acquisition, Writing – review & editing. AH-B: Funding acquisition, Writing – review & editing. BR-P: Data curation, Formal analysis, Writing – review & editing. RV-C: Conceptualization, Funding acquisition, Supervision, Writing – original draft, Writing – review & editing.
