# Supplementary material for: Interaction between MARK3 (rs11623869), PLCB4 (rs6086746) and GEMIN2 (rs2277458) variants with bone mineral density and serum 25-hidroxivitamin D levels in Mexican Mestizo women
Source: Front Endocrinol (Lausanne). 2024 Apr 23;15:1392063. doi: 10.3389/fendo.2024.1392063 (PMC11074919; doi:10.3389/fendo.2024.1392063)
Supplement: Supplementary file 2 [file Table_1.docx]

Supplementary Material

*Interaction between MARK3 (*rs11623869)*, PLCB4* (rs6086746) *and GEMIN2* (rs2277458) *Variants with bone mineral density and serum 25-hidroxivitamin D levels in Mexican Mestizo women..*

Diana I Aparicio-Bautista^1,†^, Rogelio F Jiménez-Ortega^1,2,†^, Adriana Becerra-Cervera^1,3^, Arnoldo Aquino-Galvez^4^, Valeria Ponce de León-Suárez^5^, Leonora Casas-Ávila^5^, Jorge Salmerón^6^, Alberto Hidalgo-Bravo^5^, Berenice Rivera-Paredez^6^, and Rafael Velázquez-Cruz^1*^.

*** Correspondence:** Rafael Velázquez Cruz: [rvelazquez@inmegen.gob.mx](mailto:rvelazquez@inmegen.gob.mx)

| **Supplementary Table 1. Sociodemographic and clinical characteristics of the study population.** | | | | |
| --- | --- | --- | --- | --- |
|  | **Total women** | **Pre-menopausal** | **Post-menopausal** |  |
|  | **n=1,300** | **n=521** | **n=779** | **P value** |
| Age, years^a^ | 54(43-63) | 41(31-47) | 61(55-67) | <0.001 |
| BMI^a^ | 26.8(24.1-30.3) | 25.4(23-29.2) | 27.4(24.9-30.8) | <0.001 |
| Overweitgh,% | 39.9 | 33.8 | 44.0 | 0.0002 |
| Obesity,% | 26.2 | 20.2 | 30.3 | <0.001 |
| Waist circumference,cm^a^ | 92(85-100) | 89(82-97) | 95(88-102) | <0.001 |
| Body fat proportion^a^ | 45.0(40.7-49.2) | 43.9(39.4-48.2) | 45.7(42.0-49.8) | <0.001 |
| Fasting glucose, mg/dl^a^ | 96(90-105) | 93(87-100) | 99(92-109) | <0.001 |
| Impaired,% | 5.8 | 2.5 | 8.0 | <0.001 |
| Diabetes,% | 16.8 | 9.6 | 21.6 | <0.001 |
| Triglycerides, mg/dl^a^ | 151(110-202) | 137(97-186) | 162(118-211) | <0.001 |
| Elevated,% | 50.6 | 42.8 | 55.8 | <0.001 |
| HDL, mg/dl^a^ | 46(39-54) | 46(39-53) | 46(39-54) | 0.5042 |
| Low,% | 64.4 | 67.0 | 62.6 | 0.1045 |
| LDL, mg/dl^a^ | 121(99-147) | 112(95-135) | 128(104-154) | <0.001 |
| Elevated,% | 74.5 | 66.7 | 79.7 | <0.001 |
| Cholesterol, mg/dl^a^ | 199(172-227) | 191(165-214) | 206(178-236) | <0.001 |
| Elevated,% | 48.9 | 38.8 | 55.7 | <0.001 |
| ALT | 20(15-29) | 19(14-26) | 21(16-30) | <0.001 |
| AST | 23(20-30) | 22(18-27) | 25(21-31) | <0.001 |
| Elevated, % (AST/ALT > 40) | 13.8 | 11.7 | 15.2 | <0.001 |
| 25-hydroxivitamin D | 20.9(16.8-24.7) | 20.5(16.2-24.6) | 21(17.1-24.9) | 0.0845 |
| VD deficiency, % | 44.3 | 47.8 | 42.0 | 0.0391 |
| Total energy,kcal/day^a^ | 1696(1261-2233) | 1729(1312-2320) | 1680(1222-2146) | 0.0424 |
| Total hip BMD, g/cm^2 a^ | 0.964(0.871-1.072) | 1.027(0.933-1.122) | 0.925(0.840-1.017) | <0.001 |
| Low-hip BMD, % | 27.9 | 13.4 | 37.4 | <0.001 |
| Femoral neck BMD, g/cm^2a^ | 0.932(0.830-1.027) | 1.005(0.921-1.095) | 0.874(0.790-0.964) | <0.001 |
| Low-femoral neck BMD, % | 42.1 | 20.0 | 57.0 | <0.001 |
| Lumbar spine BMD, g/cm^2a^ | 1.068(0.950-1.174) | 1.152(1.070-1.241) | 0.999(0.896-1.104) | <0.001 |
| Low-lumbar spine BMD, % | 53.1 | 27.5 | 70.4 | <0.001 |
| ^a^ Median (P25-P75) **p*-value obtained with Wilcoxon rank-sum test, Student's t-test, or chi-square test, as appropriate. | | | | |

| **Supplementary Table 2.** **Sociodemographic and clinical characteristics of the study population by rs11623869 genotypes in total women.** | | | | | | |
| --- | --- | --- | --- | --- | --- | --- |
|  | **GG** | **GT** | **TT** | **GG** | **GT+TT** |  |
|  | **n=833** | **n=407** | **n=46** | **n=833** | **n=453** | **P value** |
| Age, years^a^ | 54(43-62) | 54(44-64) | 55(46-62) | 54(43-62) | 54(44-64) | 0.0871 |
| BMI^a^ | 26.8(24.1-30.4) | 26.9(23.6-29.9) | 27.1(24.2-30.1) | 26.8(24.1-30.4) | 26.9(23.7-30) | 0.5990 |
| Overweitgh,% | 39.9 | 40.1 | 34.8 | 39.9 | 39.5 | 0.8886 |
| Obesity,% | 26.7 | 25.1 | 30.4 | 26.7 | 25.6 | 0.6686 |
| Waist circumference,cm^a^ | 93(85-100) | 92(85-101) | 90(84-101) | 93(85-100) | 92(85-101) | 0.5156 |
| Body fat proportion^a^ | 44.9(40.8-49.7) | 45.1(41.0-48.5) | 46.2(40.3-50.7) | 44.9(40.8-49.7) | 45.1(40.8-48.7) | 0.7113 |
| Fasting glucose, mg/dl^a^ | 96(90-105) | 96(90-105) | 95(89-105) | 96(90-105) | 96(90-104) | 0.8411 |
| Impaired,% | 5.3 | 6.9 | 4.4 | 5.3 | 6.6 | 0.3390 |
| Diabetes,% | 16.6 | 17.0 | 19.6 | 16.6 | 17.2 | 0.7834 |
| Triglycerides, mg/dl^a^ | 153(110-203) | 148(108-196) | 163(120-223) | 153(110-203) | 151(110-199) | 0.7083 |
| Elevated,% | 50.8 | 50.1 | 58.7 | 50.8 | 51.0 | 0.9454 |
| HDL, mg/dl^a^ | 46(39-54) | 46(39-54) | 46(39-51) | 46(39-54) | 46(39-54) | 0.9589 |
| Low,% | 63.9 | 64.6 | 71.7 | 63.9 | 65.4 | 0.5914 |
| LDL, mg/dl^a^ | 120(98-145) | 124(103-150) | 120(89-166) | 120(98-145) | 123(103-151) | 0.0248 |
| Elevated,% | 72.7 | 79.2 | 64.4 | 72.7 | 77.7 | 0.0495 |
| Cholesterol, mg/dl^a^ | 197(171-225) | 202(176-231) | 195(167-251) | 197(171-225) | 201(175-234) | 0.0537 |
| Elevated,% | 47.6 | 52.1 | 47.8 | 47.6 | 51.7 | 0.1599 |
| ALT | 21(16-29) | 20(15-27) | 22(16-30) | 21(16-29) | 20(15-27) | 0.0581 |
| AST | 24(20-30) | 23(19-29) | 26(20-34) | 24(20-30) | 23(11-29) | 0.1863 |
| Elevated, % (AST/ALT > 40) | 14.5 | 12.3 | 17.4 | 14.5 | 12.8 | 0.3998 |
| 25-hydroxivitamin D | 20.7(16.5-24.6) | 21.3(17.2-25.4) | 22.1(17-24.1) | 20.7(16.5-24.6) | 21.3(17.1-25.2) | 0.0507 |
| VD deficiency, % | 45.7 | 41.5 | 41.3 | 45.7 | 41.5 | 0.1474 |
| Total energy,kcal/day^a^ | 1715(1263-2285) | 1680(1281-2186) | 1578(1137-2056) | 1715(1263-2285) | 1670(1218-2175) | 0.1249 |
| Total hip BMD, g/cm^2 a^ | 0.971(0.875-1.075) | 0.955(0.859-1.065) | 0.930(0.879-0.994) | 0.971(0.875-1.075) | 0.954(0.861-1.057) | 0.0835 |
| Low-hip BMD, % | 25.8 | 32.4 | 26.1 | 25.8 | 31.8 | 0.0219 |
| Femoral neck BMD, g/cm^2a^ | 0.936(0.835-1.029) | 0.917(0.819-1.022) | 0.913(0.837-0.986) | 0.936(0.835-1.029) | 0.916(0.819-1.020) | 0.1403 |
| Low-femoral neck BMD, % | 39.9 | 46.0 | 43.5 | 39.9 | 45.7 | 0.0440 |
| Lumbar spine BMD, g/cm^2a^ | 1.071(0.960-1.174) | 1.063(0.946-1.184) | 1.058(0.896-1.117) | 1.071(0.960-1.174) | 1.062(1.175-1.061) | 0.4266 |
| Low-lumbar spine BMD, % | 52.2 | 53.3 | 63.0 | 52.2 | 54.3 | 0.4710 |
| ^a^ Median (P25-P75), **p*-value for GG vs GT+TT obtained with Wilcoxon rank-sum test, Student's t-test, or chi-square test, as appropriate. | | | | | | |

| **Supplementary Table 3. Sociodemographic and clinical characteristics of the study population by rs6086746 genotypes in total women.** | | | | | | |
| --- | --- | --- | --- | --- | --- | --- |
|  | **GG** | **GA** | **AA** | **GG** | **GA+AA** |  |
|  | **n=693** | **n=503** | **n=79** | **n=693** | **n=582** | ***p*-value*** |
| Age, years^a^ | 54(44-63) | 53(42-63) | 54(43-61) | 54(44-63) | 54(43-63) | 0.4190 |
| BMI^a^ | 26.8(24.1-30.1) | 26.8(23.8-30.3) | 26.9(23.8-30.4) | 26.8(24.1-30.1) | 26.8(23.8-30.4) | 0.7311 |
| Overweitgh,% | 41.5 | 38.4 | 32.9 | 41.5 | 37.6 | 0.1562 |
| Obesity,% | 25.9 | 26.4 | 29.1 | 25.9 | 26.8 | 0.7161 |
| Waist circumference,cm^a^ | 92(85-100) | 93(85-102) | 92(85-97) | 92(85-100) | 93(85-101) | 0.6133 |
| Body fat proportion^a^ | 44.9(41.1-49.3) | 45.2(40.7-48.9) | 44.5(40.2-49.0) | 44.9(41.1-49.3) | 45.1(40.6-48.9) | 0.8057 |
| Fasting glucose, mg/dl^a^ | 97(90-106) | 95(90-103) | 95(88-107) | 97(90-106) | 95(89-104) | 0.0453 |
| Impaired,% | 6.6 | 4.4 | 8.9 | 6.6 | 5.0 | 0.2259 |
| Diabetes,% | 17.4 | 15.9 | 15.2 | 17.4 | 15.8 | 0.4450 |
| Triglycerides, mg/dl^a^ | 158(113-207) | 141(106-191) | 159(115-215) | 158(113-207) | 143(106-194) | 0.0057 |
| Elevated,% | 54.9 | 44.9 | 51.9 | 54.9 | 45.9 | 0.0014 |
| HDL, mg/dl^a^ | 45(39-53) | 46(39-54) | 45(40-53) | 45(39-53) | 46(40-54) | 0.0759 |
| Low,% | 66.9 | 61.0 | 65.8 | 66.9 | 61.7 | 0.0531 |
| LDL, mg/dl^a^ | 121(98-147) | 119(98-146) | 123(106-148) | 122(98-147) | 120(99-146) | 0.9666 |
| Elevated,% | 74.1 | 73.5 | 82.1 | 74.4 | 74.6 | 0.9349 |
| Cholesterol, mg/dl^a^ | 199(172-229) | 196(169-226) | 200(181-231) | 199(172-229) | 197(171-226) | 0.4835 |
| Elevated,% | 49.9 | 46.9 | 50.6 | 49.9 | 47.4 | 0.3735 |
| ALT | 20(15-28) | 20(15-29) | 21(16-31) | 20(15-28) | 20(15-29) | 0.6817 |
| AST | 23(20-30) | 24(19-30) | 24(19-28) | 23(20-30) | 24(19-29) | 0.5688 |
| Elevated, % (AST/ALT > 40) | 14.7 | 13.7 | 10.1 | 14.7 | 13.2 | 0.4420 |
| 25-hydroxivitamin D | 20.7(16.5-24.6) | 21.3(17.2-25.4) | 22.1(17-24.1) | 20.7(16.5-24.6) | 21.3(17.1-25.2) | 0.0507 |
| VD deficiency, % | 45.7 | 42.2 | 44.3 | 45.7 | 42.4 | 0.2371 |
| Total energy,kcal/day^a^ | 1690(1282-2194) | 1688(1192-2255) | 1806(1477-2300) | 1690(1282-2194) | 1703(1228-1843) | 0.9627 |
| Total hip BMD, g/cm^2 a^ | 0.954(0.860-1.058) | 0.974(0.879-1.080) | 1.000(0.887-1.100) | 0.954(0.860-1.058) | 0.977(0.882-1.080) | 0.0051 |
| Low-hip BMD, % | 30.4 | 25.3 | 24.1 | 30.4 | 25.1 | 0.0357 |
| Femoral neck BMD, g/cm^2a^ | 0.920(0.821-1.020) | 0.941(0.835-1.041) | 0.956(0.848-1.065) | 0.920(0.821-1.020) | 0.942(0.839-1.043) | 0.0131 |
| Low-femoral neck BMD, % | 45.1 | 38.4 | 34.2 | 45.1 | 37.1 | 0.0039 |
| 3 | 1.049(0.929-1.159) | 1.091(0.983-1.200) | 1.081(0.991-1.174) | 1.049(0.929-1.159) | 1.089(0.985-1.200) | 0.00003 |
| ^a^ Median (P25-P75), **p*-value for GG vs GT+TT obtained with Wilcoxon rank-sum test, Student's t-test, or chi-square test, as appropriate. | | | | | | |

| **Supplementary Table 4. Sociodemographic and clinical characteristics of the study population by rs2277458 in total women.** | | | | | | |
| --- | --- | --- | --- | --- | --- | --- |
|  | **GG** | **GA** | **AA** | **GG** | **GA+AA** |  |
|  | **n=692** | **n=513** | **n=85** | **n=692** | **n=598** | ***p*-value*** |
| Age, years^a^ | 54(43-63) | 54(44-63) | 53(43-62) | 54(43-63) | 54(43-63) | 0.9630 |
| BMI^a^ | 27.1 (24.1-30.1) | 26.6(24.0-30.5) | 25.6(23.8-29.3) | 27.1 (24.1-30.1) | 26.5(24.0-30.3) | 0.5532 |
| Overweitgh,% | 40.9 | 38.0 | 42.4 | 40.9 | 38.6 | 0.2881 |
| Obesity,% | 26.0 | 27.3 | 21.2 | 26.0 | 26.4 | 0.8706 |
| Waist circumference,cm^a^ | 93(86-100) | 92(85-100) | 92(84-101) | 93(86-100) | 92(84-101) | 0.3509 |
| Body fat proportion^a^ | 44.7(40.7-44.6) | 45.5(40.9-49.6) | 44.1(38.7-49.1) | 44.7(40.7-44.6) | 45.3(40.8-49.5) | 0.4713 |
| Fasting glucose, mg/dl^a^ | 96.5(90-105) | 95(90-105) | 95(90-102) | 96.5(90-105) | 95(90-105) | 0.3131 |
| Impaired,% | 5.8 | 6.6 | 1.2 | 5.8 | 5.9 | 0.9391 |
| Diabetes,% | 16.2 | 17.9 | 14.1 | 16.2 | 17.4 | 0.5650 |
| Triglycerides, mg/dl^a^ | 154(107-205) | 147(113-192) | 167(120-215) | 154(107-205) | 149(115-194) | 0.7801 |
| Elevated,% | 51.6 | 48.3 | 57.7 | 51.6 | 49.7 | 0.4961 |
| HDL, mg/dl^a^ | 45.1(39-53.5) | 45.8(39-53.6) | 47.6(40.9-56) | 45.1(39-53.5) | 46(39-54) | 0.6565 |
| Low,% | 65.6 | 63.6 | 60.0 | 65.6 | 63.0 | 0.3308 |
| LDL, mg/dl^a^ | 121(99-147) | 121(99-148) | 124(96-144) | 121(99-147) | 121(99-147) | 0.7858 |
| Elevated,% | 74.4 | 73.9 | 69.4 | 74.4 | 73.2 | 0.6248 |
| Cholesterol, mg/dl^a^ | 199(172-230) | 199(172-227) | 200(172-223) | 199(172-230) | 199(172-227) | 0.6599 |
| Elevated,% | 48.4 | 49.7 | 50.6 | 48.4 | 49.8 | 0.6160 |
| ALT | 20(15-28) | 20(15-29) | 20(16-28) | 20(15-28) | 20(15-29) | 0.7235 |
| AST | 23(20-29) | 24(19-30) | 24(20-29) | 23(20-29) | 24(19-30) | 0.6835 |
| Elevated, % (AST/ALT > 40) | 13.7 | 13.8 | 15.3 | 13.7 | 14.1 | 0.8359 |
| 25-hydroxivitamin D | 20.6(16.7-24.4) | 20.9(16.6-25.1) | 21.7(18.1-25.1) | 20.6(16.7-24.4) | 21.1(16.8-25.1) | 0.1071 |
| VD deficiency, % | 45.4 | 44.1 | 37.7 | 45.4 | 43.1 | 0.4070 |
| Total energy,kcal/day^a^ | 1650(1256-2179) | 1767(1267-2299) | 1689(1249-2139) | 1650(1256-2179) | 1755(1262-2282) | 0.1181 |
| Total hip BMD, g/cm^2 a^ | 0.963(0.875-1.075) | 0.967(0.867-1.064) | 0.972(0.856-1.062) | 0.963(0.875-1.075) | 0.967(0.864-1.064) | 0.4874 |
| Low-hip BMD, % | 26.7 | 28.5 | 32.9 | 26.7 | 29.10 | 0.3374 |
| Femoral neck BMD, g/cm^2a^ | 0.931(0.833-1.027) | 0.929(0.825-1.025) | 0.946(0.819-1.029) | 0.931(0.833-1.027) | 0.933(0.824-1.025) | 0.6211 |
| Low-femoral neck BMD, % | 41.9 | 43.1 | 36.5 | 41.9 | 42.1 | 0.9421 |
| Lumbar spine BMD, g/cm^2a^ | 1.071(0.947-1.181) | 1.056(0.953-1.163) | 1.059(0.984-1.181) | 1.071(0.947-1.181) | 1.056(0.956-1.168) | 0.4077 |
| Low-lumbar spine BMD, % | 51.7 | 54.2 | 56.5 | 51.7 | 54.5 | 0.3150 |
| ^a^ Median (P25-P75) **p*-value for GG vs GT+TT obtained with Wilcoxon rank-sum test, Student's t-test, or chi-square test, as appropriate. | | | | | | |

| **Supplementary table 5**. **Association between the variants of interest and low-BMD at different sites and VD deficiency among women aged 45 years and older.** | | | | | | | | | |
| --- | --- | --- | --- | --- | --- | --- | --- | --- | --- |
|  |  | **Total hip BMD** | | **Femoral neck BMD** | | **Lumbar spine BMD** | | **VD Deficiency*** | |
| rs11623869 |  | **OR (95%CI)** | ***p*-value** | **OR (95%CI)** | ***p*-value** | **OR (95%CI)** | ***p*-value** | **OR (95%CI)** | ***p*-value** |
| Additive |  | 1.07  (0.80-1.44) | 0.644 | 1.10  (0.82-1.45) | 0.529 | 0.94  (0.72-1.23) | 0.659 | 0.78  (0.61-1.01) | 0.057 |
| Codominant | GG | 1.0 |  | 1.0 |  | 1.0 |  | 1.0 |  |
|  | GT | 1.21  (0.85-1.72) | 0.290 | 1.07  (0.76-1.50) | 0.694 | 0.92  (0.67-1.26) | 0.600 | 0.76  (0.56-1.02) | 0.068 |
|  | TT | 0.77  (0.30-2.01) | 0.600 | 1.30  (0.54-3.14) | 0.552 | 0.97  (0.43-2.22) | 0.947 | 0.70  (0.31-1.55) | 0.374 |
| Dominant | GG | 1.0 |  | 1.0 |  | 1.0 |  | 1.0 |  |
|  | GT+TT | 1.16  (0.83-1.63) | 0.390 | 1.09  (0.79-1.51) | 0.604 | 0.92  (0.68-1.25) | 0.611 | 0.73  (0.56-1.00) | 0.053 |
| Recesive | GG+GT | 1.0 |  | 1.0 |  | 1.0 |  | 1.0 |  |
|  | TT | 0.73  (0.28-1.87) | 0.508 | 1.27  (0.53-3.04) | 0.584 | 1.02  (0.44-2.27) | 0.997 | 0.76  (0.34-1.69) | 0.506 |
| rs6086746 |  |  |  |  |  |  |  |  |  |
| Additive |  | 0.68  (0.52-0.91) | 0.010 | 0.69  (0.53-0.90) | 0.006 | 0.68  (0.54-0.88) | 0.003 | 0.83  (0.66-1.05) | 0.119 |
| Codominant | GG | 1.0 |  | 1.0 |  | 1.0 |  | 1.0 |  |
|  | GA | 0.65  (0.46-0.92) | 0.016 | 0.65  (0.46-0.91) | 0.011 | 0.62  (0.46-0.85) | 0.003 | 0.77  (0.57-1.02) | 0.071 |
|  | AA | 0.55  (0.25-1.19) | 0.128 | 0.55  (0.28-1.10) | 0.090 | 0.59  (0.31-1.10) | 0.098 | 0.84  (0.46-1.53) | 0.565 |
| Dominant | GG | 1.0 |  | 1.0 |  | 1.0 |  | 1.0 |  |
|  | GA+AA | 0.63  (0.45-0.89) | 0.009 | 0.63  (0.46-0.87) | 0.006 | 0.62  (0.46-0.83) | 0.002 | 0.77  (0.59-1.02) | 0.072 |
| Recesive | GG+GA | 1.0 |  | 1.0 |  | 1.0 |  | 1.0 |  |
|  | AA | 0.65  (0.31-1.40) | 0.275 | 0.66(0.34-1.29) | 0.226 | 0.72  (0.39-1.34) | 0.301 | 0.94  (0.52-1.70) | 0.838 |
| rs2277458 |  |  |  |  |  |  |  |  |  |
| Additive |  | 1.05  (0.81-1.38) | 0.692 | 0.80  (0.62-1.04) | 0.103 | 1.10  (0.86-1.40) | 0.440 | 0.82  (0.66-1.03) | 0.086 |
| Codominant | GG | 1.0 |  | 1.0 |  | 1.0 |  | 1.0 |  |
|  | GA | 1.09  (0.77-1.53) | 0.636 | 0.96  (0.69-1.33) | 0.788 | 1.02  (0.75-1.39) | 0.892 | 0.86  (0.65-1.14) | 0.298 |
|  | AA | 1.05  (0.52-2.11) | 0.887 | 0.45  (0.23-0.90) | 0.023 | 1.43  (0.74-2.75) | 0.282 | 0.61  (0.34-1.11) | 0.298 |
| Dominant | GG | 1.0 |  | 1.0 |  | 1.0 |  | 1.0 |  |
|  | GA+AA | 1.08  (0.78-1.51) | 0.641 | 0.87  (0.63-1.19) | 0.375 | 1.07  (0.79-1.43) | 0.669 | 0.82  (0.62-1.08) | 0.158 |
| Recesive | GG+GA | 1.0 |  | 1.0 |  | 1.0 |  | 1.0 |  |
|  | AA | 1.01  (0.51-2.00) | 0.966 | 0.46  (0.24-0.90) | 0.024 | 1.42  (0.75-2.69) | 0.285 | 0.65  (0.37-1.17) | 0.151 |
| Model adjusted for age (years), BMI categories, energy intake, calcium intake (tertiles), vitamin D intake (tertiles), calcium supplementation, alcohol consumption (g/day), smoking status (never, current and past), physical activity, and hormone replacement therapy (HRT). Low-BMD as a T-score below -1 at the total hip, and lumbar spine. *Model adjusted for age (years), BMI categories, energy intake, vitamin D intake (tertiles), alcohol consumption (g/day), smoking status (never, current and past), physical activity, blood collection season, and hormone replacement therapy (HRT). | | | | | | | | | |
